# Subclinical cardiac involvement during diabetic ketoacidosis at type 1 diabetes onset in youth: biomarker elevation, altered diastolic filling, and reduced global longitudinal strain: a prospective cohort study

**DOI:** 10.1186/s12933-026-03377-9

**Published:** 2026-09-18

**Authors:** Jantje Weiskorn, Sophia Hotze, Alexander Freiherr von Gise, Marlene Busse, Olga Kordonouri

**Affiliations:** AUF DER BULT Children’s Hospital, Janusz-Korczak-Allee 12, 30173 Hannover, Germany

**Keywords:** T1D, DKA, Pediatric cardiology

## Abstract

**Background:**

Diabetic ketoacidosis (DKA) may be associated with cardiac involvement. Increased cardiac biomarkers and abnormalities in cardiac function were mainly reported in adults with type 1 diabetes (T1D). Pediatric data are lacking. This study therefore assessed cardiac biomarker alterations and echocardiographic changes, including strain analysis, in children with diabetic ketoacidosis at the onset of type 1 diabetes.

**Research design and methods:**

In this prospective, observational, cohort study, patients < 18 years with T1D onset were consecutively enrolled at a single center from January 2024 to March 2025 and classified as DKA or non-DKA. Cardiac biomarkers (troponin I, NT-proBNP) were measured before and 24 h after therapy initiation in all patients, and additionally at 48 and 72 h in those with DKA. Echocardiography, including speckle-tracking strain analysis, was performed after 24 h in both groups and after 6 days in the DKA group.

**Results:**

Eighty-one patients were included (60% male; median age 9.8 years [5.2–14.1]); 43 (53.1%) had DKA. Troponin I and NT-proBNP were significantly higher in the DKA group at baseline (troponin I: 5.7 [4.1–10.8] vs. 3.9 [3.9–4.6] ng/L, *p* < 0.001; NT-proBNP: 87.0 [41.0–231.0] vs. 40.0 [16.8–69.0] ng/L, *p* = 0.002) and remained elevated at 24 h. Echocardiography showed lower E/A ratios, higher peak a′-wave velocities, and a significantly reduced left ventricular global longitudinal strain (GLS) in the DKA group (−  19.2 [−  20.4 to − 18.1] vs. − 21.5 [−  23.0 to − 18.9], *p* = 0.029), with most values within the normal range. In multivariable analysis, lower bicarbonate (indicating greater DKA severity) was independently associated with higher troponin I and NT-proBNP, and younger age with higher NT-proBNP.

**Conclusions:**

In children with T1D, DKA at onset is associated with subclinical cardiac involvement, reflected by elevated cardiac biomarkers, altered diastolic filling parameters, and reduced left ventricular GLS. These findings indicate myocardial stress during the acute phase of DKA; whether they carry prognostic significance requires studies with longer-term follow-up.

**Trial registration:**

The study was registered in the German Clinical Trial Register (DRKS00032719).

**Graphical abstract:**

**Figure:**
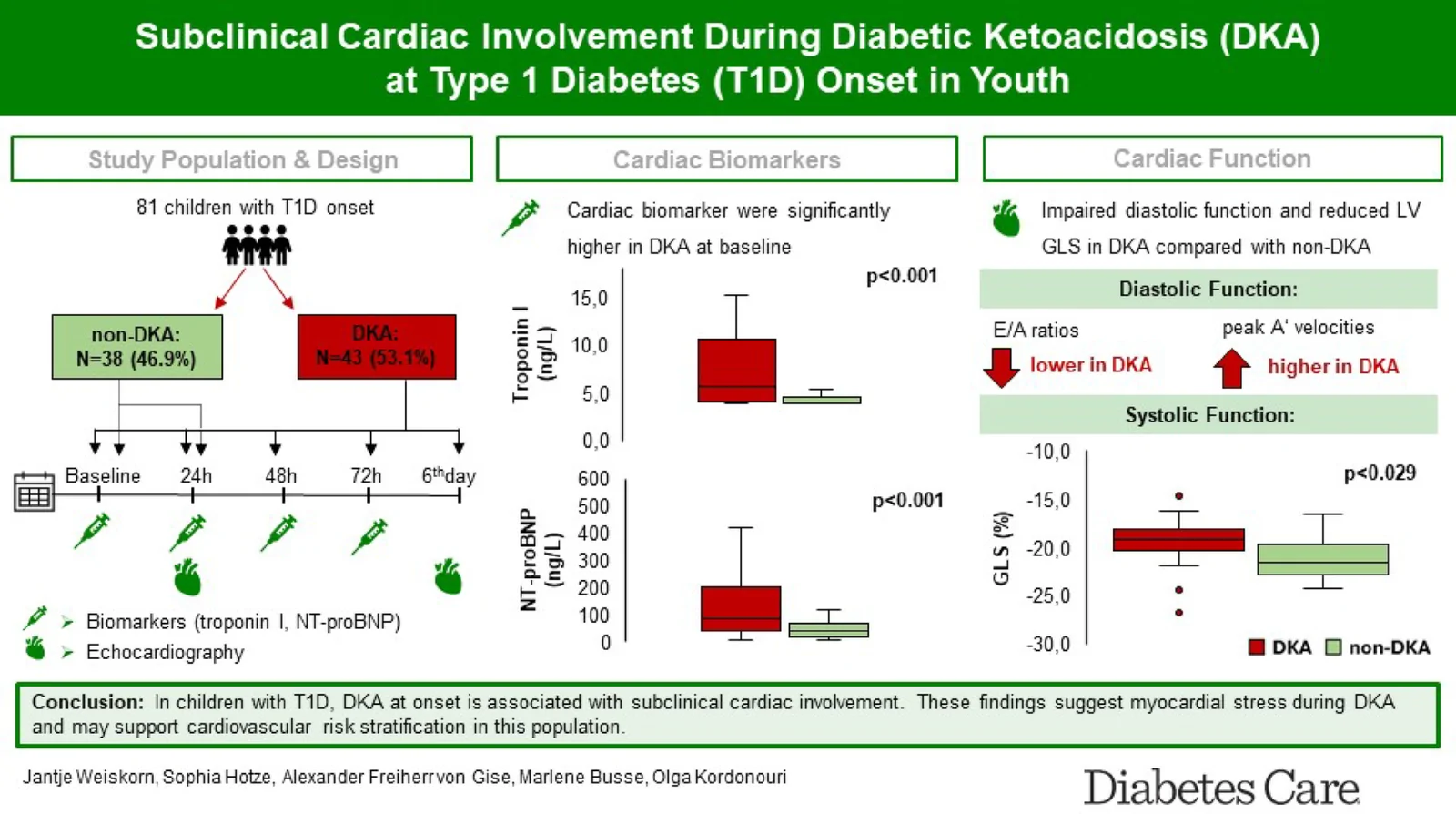

**Supplementary Information:**

The online version contains supplementary material available at https://doi.org/10.1186/s12933-026-03377-9.

**Research highlights**

**What is currently known about this topic?**

DKA at T1D onset may cause cardiac stress, but pediatric data combining cardiac biomarkers and strain echocardiography are lacking.

**What is the key research question?**

What is the extent and pattern of cardiac biomarker alterations and echocardiographic changes, including strain analysis, in children with DKA at the onset of T1D?

**What is new?**

Children with DKA had significantly elevated troponin I and NT-proBNP, altered diastolic filling, and reduced left ventricular global longitudinal strain (GLS) compared with non-DKA peers, with most values remaining within the normal range.

**How might this study influence clinical practice?**

DKA at T1D onset is associated with subclinical cardiac involvement, supporting acute cardiac monitoring during severe DKA and highlighting the need for longer-term follow-up.

## Background

Type 1 diabetes (T1D) is among the most common endocrine disorders in children, with an incidence of approximately 25.9 per 100,000 person-years in Germany, and represents a significant cause of pediatric morbidity [1]. Diabetic ketoacidosis (DKA) is a serious, life-threatening acute complication of T1D, occurring at disease onset in approximately 20% of children [2]. The underlying insulin deficiency leads to progressive lipolysis and subsequent ketone body formation, resulting in metabolic acidosis. This metabolic derangement can cause multi-organ dysfunction, including cerebral damage, pancreatitis, and acute kidney injury. Cardiac involvement is a plausible pathophysiological consequence of DKA due to electrolyte disturbances, metabolic acidosis, dehydration, and the catecholamine-mediated stress response.

This pathophysiological rationale is supported by clinical observations: elevated cardiac biomarkers including troponin I, creatine kinase-MB (CK-MB), and myoglobin have been reported in children and adults with DKA [3–6]. Transient troponin I elevation during DKA has been associated with poorer long-term outcomes, including higher rates of major adverse cardiac events and mortality in adults [4, 5]. N-terminal pro-B-type natriuretic peptide (NT-proBNP), a marker of myocardial wall stress and volume overload, showed reduced levels in pediatric patients at T1D onset [7], whereas elevated NT-proBNP levels have been reported in children and adults with established T1D [8, 9]. However, its role in DKA remains unclear. Additionally, transient QTc prolongations on electrocardiogram (ECG) have been associated with DKA severity [10, 11]. Echocardiographic hypercontractility, likely related to catecholamine elevation in DKA, has been observed in adults [12, 13]. Standard echocardiography has limited sensitivity for detecting subtle myocardial changes; speckle-tracking strain analysis allows more sensitive assessment of global and regional contractility. In a small cohort of 20 adults with DKA, abnormal global longitudinal strain (GLS) was identified using this technique [13].

To date, systematically collected pediatric data addressing cardiac involvement during DKA at T1D onset are lacking. Given that people with T1D face a substantially elevated long-term cardiovascular risk—associated with a lifetime loss of 17 years for girls and 14 years for boys when onset occurs before age 10 [14]—cardiac impairment at T1D onset, particularly in cases of DKA, may have an additional negative impact. The aim of this study was to characterize the extent and pattern of cardiac biomarker alterations and their correlation with echocardiographic changes at T1D onset, with a specific focus on the association of DKA severity with cardiac biomarkers and strain values.

## Research design and methods

### Study design and population

This prospective, observational, cohort study was conducted at the Children's and Youth Hospital "AUF DER BULT" in Hannover, Germany, from 1 January 2024 to 31 March 2025.

**Fig. 1:**
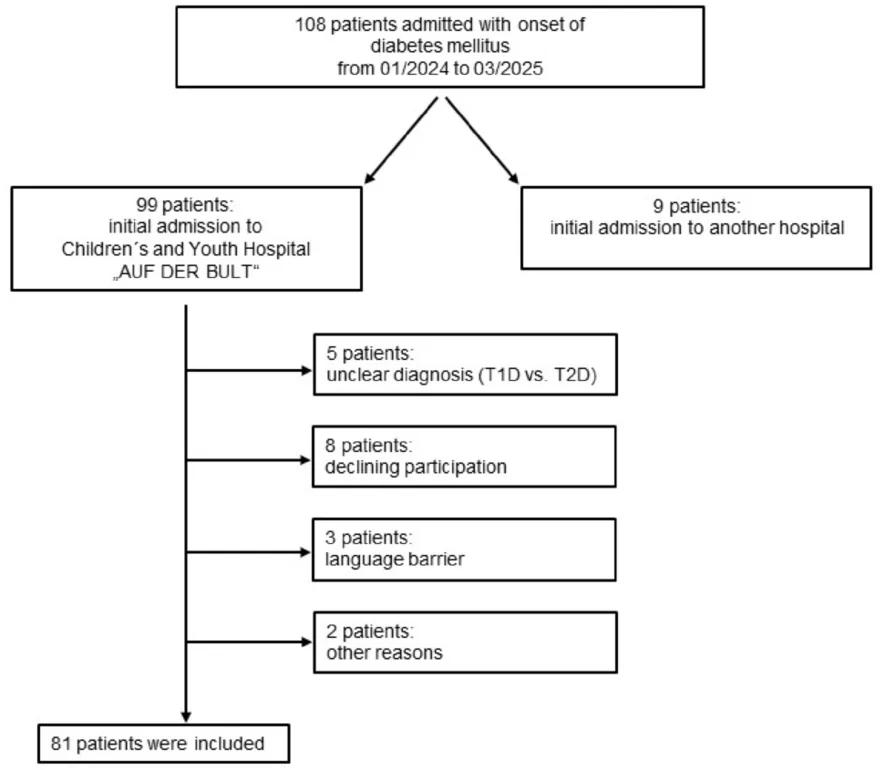
Study flow diagram. Flow chart of the study population showing enrollment and exclusion criteria for patients admitted with onset of diabetes mellitus from January 2024 to March 2025

Children and adolescents (< 18 years) with onset of T1D were included. Two cohorts were defined: participants with DKA at T1D onset (DKA group) and participants without DKA at onset (non-DKA group). Per the International Society for Pediatric and Adolescent Diabetes (ISPAD) Clinical Practice Consensus Guidelines 2022, DKA was defined as blood glucose > 200 mg/dL (> 11.1 mmol/L), venous pH < 7.3 or bicarbonate < 18 mmol/L, ketonemia (beta-hydroxybutyrate ≥ 3 mmol/L) or moderate-to-severe ketonuria. DKA severity was classified by pH: severe (pH <7.10) and mild-to-moderate (pH 7.10–7.29) [15]. Exclusion criteria were known congenital heart defects, pre-existing heart failure, known arterial hypertension, renal insufficiency, chronic pulmonary disease, malignancy, and pregnancy. All children and adolescents consecutively admitted with new-onset diabetes mellitus during the study period were screened for eligibility. Patients whose first admission was to another hospital were not eligible. Among those initially admitted to our center, individuals were not enrolled because of a language barrier precluding informed consent, declined participation, an initially unclear diabetes classification (type 1 versus type 2), or other reasons. Eligible participants were enrolled after written informed consent and assigned to the DKA or non-DKA group according to their acid–base status at presentation. The screening, exclusion, and enrollment process is summarized in Fig. 1.

### Laboratory parameters

Demographic and anthropometric data (age, sex, weight, height, BMI, and BMI standard deviation score [BMI-SDS] per Kromeyer-Hauschild et al. [16]) were recorded on admission. Clinical (heart rate, systolic and diastolic blood pressure) and laboratory parameters — islet autoantibodies (zinc transporter 8 autoantibody, glutamic acid decarboxylase autoantibody, islet antigen-2 autoantibody, and insulin autoantibody), electrolytes, blood gases (pH, bicarbonate, base excess [BE], pCO2), glucose, osmolality, creatinine, troponin I, and NT-proBNP—were measured in all patients before therapy initiation (baseline) and 24 ± 6 h after admission. Cardiac biomarkers were measured from serum samples using a chemiluminescent microparticle immunoassay (CMIA; Abbott). The pathological thresholds were defined as troponin I greater than 60 ng/L and NT-proBNP greater than 200 ng/L for children and adolescents beyond the neonatal period. C-reactive protein (CRP) and glycated hemoglobin (HbA1c; reported as both % and mmol/mol) were measured at baseline. In patients with DKA, measurements were repeated at 48 and 72 h after admission. The number of hypokalemia episodes (potassium < 3.5 mmol/L) and hypoglycemia episodes (glucose < 70 mg/dL [< 3.9 mmol/L]) were recorded within 24-h intervals, as was daily infusion volume at 24 and 48 h [17].

### Echocardiographic parameters

A 12-lead ECG was recorded using the Amedtec ECGpro CardioPart 12 USB-s device 24 ± 6 h after admission in all patients. Echocardiography was performed 24 ± 6 h after admission in all patients, and again after 6 ± 1 days in the DKA group, using a Philips Affinity 70G ultrasound device with TOMTEC AutoStrain LV and RV (Affinity Rev. 9.0 SW).

Standard echocardiographic parameters included: Simpson's ejection fraction (EF), fractional area change of the left ventricle (FAC LV), Bullet method left ventricular ejection fraction (Bullet LV EF), Teichholz ejection fraction, aortic valve velocity–time integral (AV VTI), tricuspid annular plane systolic excursion (TAPSE) and mitral annular plane systolic excursion (MAPSE) as parameters of systolic function. Tricuspid valve E/A ratio (TV E/A) and mitral valve E/A ratio (MV E/A) were included as parameters of diastolic function. Tissue Doppler imaging yielded medial s′-peak velocity (Med s′ Vmax), lateral s′-peak velocity (Lat s′ Vmax), right ventricular s′-peak velocity (RV s′ Vmax), medial e′-peak and a′-peak velocities (Med e′ Vmax, Med a′ Vmax), lateral e′-peak and a′-peak velocities (Lat e′ Vmax, Lat a′ Vmax), e′/a′ medial, e′/a′ lateral, and right ventricular e′-peak and a′-peak velocities with RV e′/a′. Ventricular dimensions and wall thickness measurements included right ventricular anterior wall thickness in diastole (RVAWd), right ventricular end-diastolic diameter (RVDD), left ventricular end-diastolic diameter (LVEDD), left ventricular end-systolic diameter (LVESD), left ventricular posterior wall thickness in diastole and systole (LVPWd, LVPWs), and interventricular septal thickness in diastole and systole (IVSd, IVSs); z-scores were calculated per Kampmann et al. [18]. Speckle-tracking strain values were obtained in the four-chamber view for the right ventricle (RV A4C) and left ventricle (LV A4C), and GLS was measured for the left ventricle; z-scores for RV A4C and LV A4C were derived per Romanowicz et al. [19].

### Statistical analysis

Statistical analyses were performed using IBM SPSS Statistics, version 29 (IBM Corp., Armonk, NY, USA). Continuous variables were tested for normality using the Kolmogorov–Smirnov and Shapiro–Wilk tests and expressed as median with interquartile range (IQR). Group comparisons used the Mann–Whitney U test for continuous variables and the chi-square test for categorical variables. Paired comparisons used the Wilcoxon signed-rank test; comparisons across more than two time points used the Friedman test. Spearman's rho was used for correlation analyses. Because troponin I and NT-proBNP were strongly right-skewed, both were natural log-transformed for regression analyses. To assess whether biomarker elevations were independent of potential confounders, prespecified multivariable linear regression models were fitted with log-transformed baseline troponin I and log-transformed baseline NT-proBNP as dependent variables and baseline age, HbA1c, serum osmolality, glucose, and bicarbonate as covariates, with bicarbonate serving as the index of DKA severity. Creatinine was not included as a covariate because it is age- and muscle-mass-dependent, was not independently associated with either biomarker, and increased multicollinearity. Model assumptions and influential observations were assessed using standardized and studentized deleted residuals, leverage values, Cook's distance, variance inflation factors, and collinearity diagnostics. As a sensitivity analysis, the models were refitted after excluding a single influential observation (Cook's distance > 1). Two-sided *p* values < 0.05 were considered statistically significant. Given the exploratory nature of this study and the limited sample size, no formal subgroup analyses or tests for interaction were performed; all correlation analyses should be considered hypothesis-generating. Accordingly, DKA severity was analyzed primarily as a continuous measure (bicarbonate and pH); the pH-based severity categories were used for descriptive classification only. Missing data were handled by complete-case analysis; no imputation was performed, and the number of available observations is indicated for each parameter in the tables. To assess whether missingness in the strain analyses was related to clinical status, participants with and without evaluable 24-h GLS were compared using the Mann–Whitney U test for continuous variables and Fisher’s exact test for categorical variables, both in the whole cohort and within the DKA group; the same approach was applied to z LV A4C as a sensitivity analysis (Supplemental Table S5).

### Sample size

The study was designed as a prospective exploratory cohort study; no formal a priori power calculation was performed. The target sample size of 55 participants with diabetic ketoacidosis (DKA) and 35 without DKA was determined empirically, based on the median annual number of children admitted with type 1 diabetes (T1D) onset to our center during the reference period 2019–2023 (median: 55 DKA cases per year out of a median of 115 total T1D manifestations per year), as prespecified in the approved study protocol. During the predefined 15-month recruitment period (January 2024–March 2025), 43 participants with DKA and 38 without DKA were enrolled, representing 78% and 109% of planned targets, respectively. Given the exploratory nature of the study, all results should be interpreted as hypothesis-generating. Findings based on smaller analytical subsets—in particular the strain analyses (GLS: n = 24 DKA vs. n=16 non-DKA)—require confirmation in larger, adequately powered prospective studies.

## Results

### Study population

Of 108 patients admitted with newly diagnosed diabetes between January 2024 and March 2025, 81 patients with T1D onset were included (60.5% male [n = 49]; median age 9.8 years [IQR 5.2–14.1]) (Fig. 1). DKA was present in 53.1% (n = 43), including 19 cases classified as severe DKA. DKA classification relied on the glycemic and acid–base criteria of the ISPAD 2022 definition (blood glucose > 200 mg/dL [> 11.1 mmol/L] and venous pH < 7.3 or bicarbonate < 18 mmol/L), which were met in all classified cases. Quantitative blood beta-hydroxybutyrate measurement was not performed as a routine diagnostic criterion at our center during the study period; ketonuria was assessed clinically but not systematically documented for study purposes. In the clinical context of new-onset T1D with hyperglycemia and metabolic acidosis of this severity, ketonemia fulfilling ISPAD criteria can be assumed to have been present in all DKA cases. The study groups did not differ significantly in sex, age, weight, BMI, or BMI-SDS (Supplemental Table S1). Islet autoantibodies were positive in 93.8% of participants (76/81), without a significant difference between the DKA and non-DKA groups (42/43 [97.7%] vs. 34/38 [89.5%]; *p* = 0.181, Fisher’s exact test); the number of positive autoantibodies also did not differ (*p* = 0.621). Because only five participants were autoantibody-negative (one DKA, four non-DKA), a formal subgroup analysis stratified by antibody status was not statistically feasible (Supplemental Table S1).

### Troponin I

**Fig. 2:**
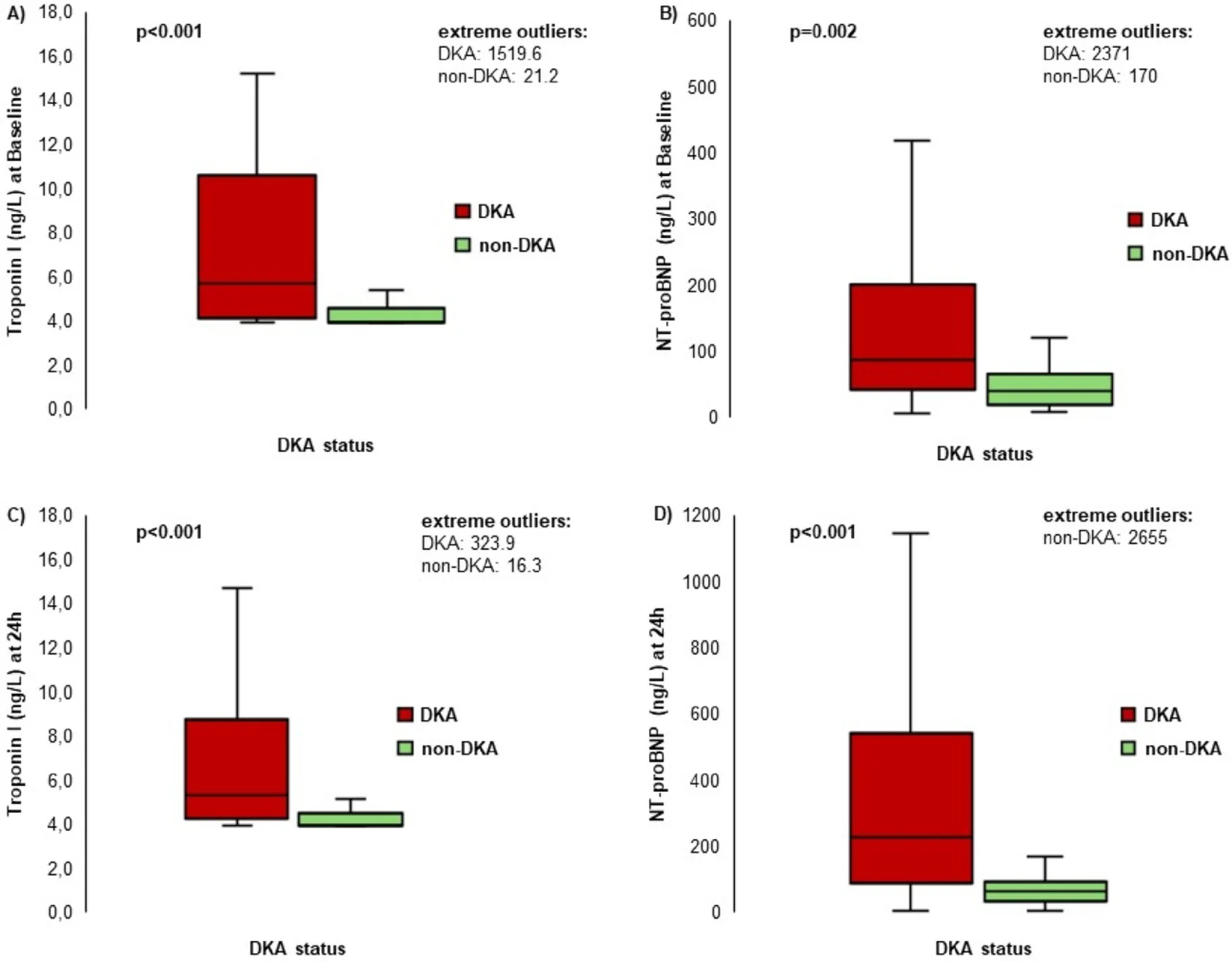
Troponin I and NT-proBNP at baseline and 24 h in DKA and Non-DKA groups. Box plots comparing cardiac biomarker levels between groups at baseline (**A**, **B**) and 24 h (**C**, **D**). Boxes represent the interquartile range (IQR) with medians indicated by horizontal lines. Whiskers indicate range excluding outliers. Extreme outliers are listed in the upper right corner of each panel. *P* values are shown in the upper left corner

**Fig. 3:**
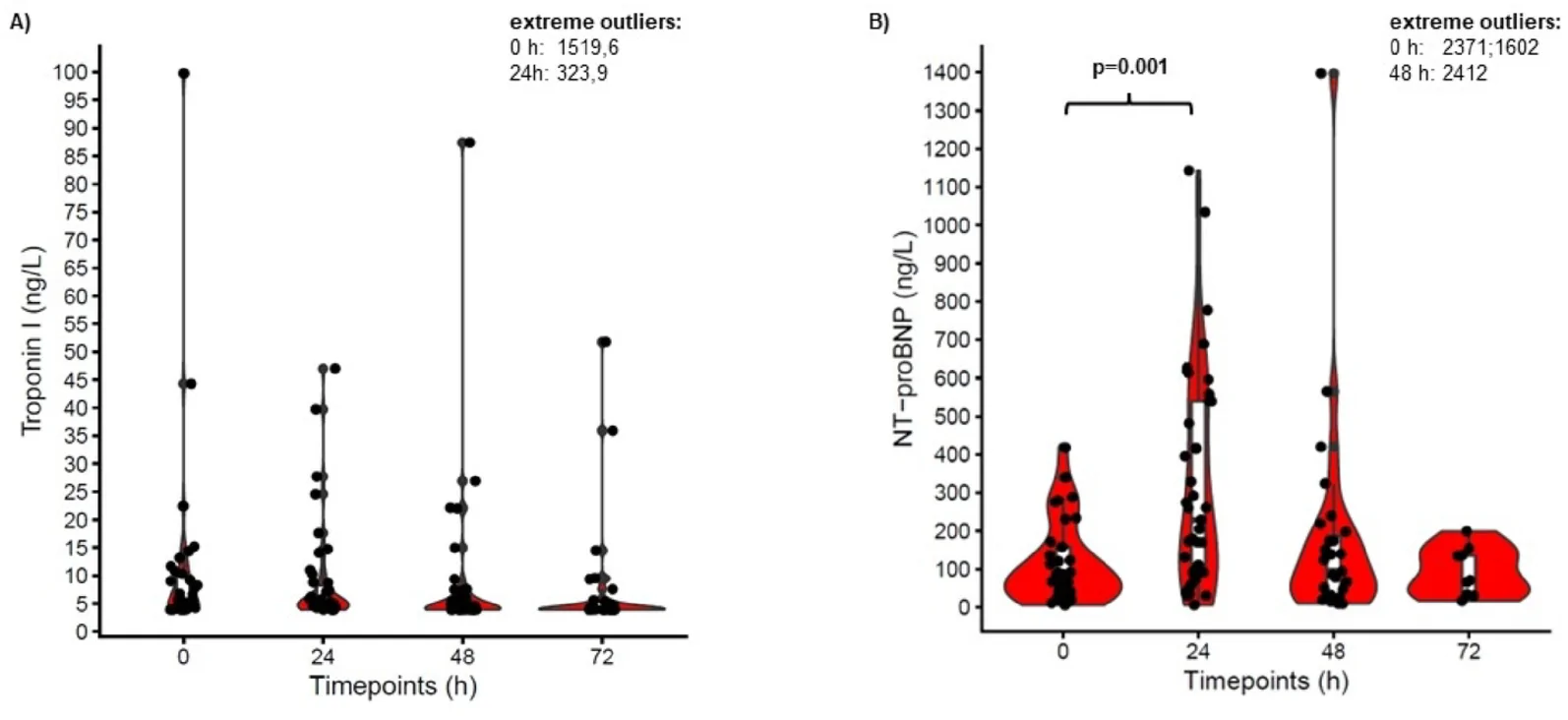
Longitudinal changes in troponin I and NT-proBNP in the DKA group. Troponin I and NT-proBNP levels in the DKA group are shown across four measurement time points (baseline, 24 h, 48 h, 72 h). Each data point represents an individual measurement. Violin plots illustrate the value distribution at each time point, with embedded box plots showing IQR and median. The bracket indicates a significant increase in NT-proBNP in the DKA group between baseline (n = 35) and 24 h (n = 43). Extreme outliers are listed in the upper right corner of each panel

Troponin I levels were significantly higher in people with DKA compared with those without DKA at baseline and after 24 h (baseline: 5.7 [4.1–10.8] vs. 3.9 [3.9–4.6] ng/L, *p* < 0.001; 24 h: 5.3 [4.2–8.8] vs. 3.9 [3.9–4.5] ng/L, *p* < 0.001) (Fig. 2). At baseline, 2 of 69 measured troponin I values (2.9%) exceeded the pathological threshold; no significant between-group difference in proportions was observed (DKA: 2/35 vs. non-DKA: 0/34). No significant changes in troponin I levels were observed across the four time points within the DKA group (Fig. 3). Baseline troponin I was significantly negatively correlated with pH (r = − 0.488, *p* < 0.001) and bicarbonate (r = − 0.482, *p* < 0.001), but not with HbA1c or initial glucose. Similar correlations were observed at 24 h (pH r = − 0.549, *p* <0.001; bicarbonate r = − 0.581, *p* < 0.001).

### NT-proBNP

NT-proBNP levels were significantly higher in the DKA group at baseline and at 24 h (baseline: 87.0 [41.0–231.0] vs. 40.0 [16.8–69.0] ng/L, *p* = 0.002; 24 h: 229.0 [88.0–539.0] vs. 63.5 [30.0–95.0] ng/L, *p* < 0.001) (Fig. 2). Pathologically elevated NT-proBNP was significantly more frequent in the DKA group (9/35 vs. 0/34, *p* = 0.002); overall, 13.0% of baseline measurements exceeded the threshold. Within the DKA group, NT-proBNP increased significantly from baseline to 24 h (87.0 [41.0–231.0] to 229.0 [88.0–539.0] ng/L; *p* < 0.001), with the 24-h median exceeding the pathological cutoff. However, the overall comparison across all four time points did not reach statistical significance (Fig. 3).

Baseline NT-proBNP correlated significantly with pH (r= − 0.443, *p* < 0.001), bicarbonate (r = − 0.461, *p* < 0.001), creatinine at 24 h (r = − 0.407, *p* = 0.001), and CRP (r = 0.292, *p* = 0.016), but not with HbA1c, initial glucose, or infusion volume during the first 24 h. NT-proBNP at 24 h showed similar correlations and was additionally associated with baseline creatinine (r = − 0.302, p = 0.009).

In prespecified multivariable linear regression adjusting for baseline age, HbA1c, serum osmolality, glucose, and bicarbonate (n = 65), lower bicarbonate was independently associated with higher log-transformed baseline troponin I (standardized β = − 0.51; B = − 0.047 per mmol/L, 95% CI − 0.068 to − 0.027; *p* < 0.001; model R^2^ = 0.35), whereas no other covariate was independently associated. For log-transformed baseline NT-proBNP, both younger age (β = − 0.43; B = − 0.087 per year, 95% CI − 0.129 to − 0.045; *p* < 0.001) and lower bicarbonate (β = − 0.43; B = − 0.067 per mmol/L, 95% CI − 0.098 to − 0.035; *p* < 0.001) were independent predictors (model R^2^ = 0.48). Influence diagnostics identified one participant with an extreme serum osmolality (385.1 mosmol/kg) as influential (Cook's distance > 1); in a sensitivity analysis excluding this case, the association of bicarbonate remained essentially unchanged for both troponin I (β = − 0.43, *p* < 0.001) and NT-proBNP (β = − 0.39, *p* < 0.001), as did the age association for NT-proBNP (β = − 0.46, *p* < 0.001). Full model coefficients are reported in Supplemental Table S3. Univariable associations of each biomarker with individual demographic, metabolic, and laboratory parameters are provided in Supplemental Table S4.

### Other laboratory parameters

As expected from the group definition, patients with DKA had significantly lower pH, bicarbonate, BE, and pCO2, and significantly higher glucose compared with non-DKA patients. No significant differences were observed in HbA1c (DKA group median 12.3% [95 mmol/mol], non-DKA 12.2% [110 mmol/mol]) or serum osmolality between the groups (Supplemental Table S1). Hypokalemia within 24 h was significantly more frequent in the DKA group (median 1 [0–4] vs. 0 [0–0] episodes, *p* = 0.001). Initial CRP was significantly elevated in the DKA group (0.29 [0.08–0.80] vs. 0.12 [0.06–0.17] mg/dL, *p* = 0.014). Creatinine did not differ at baseline but was significantly lower in the DKA group at 24 h (0.36 [0.27–0.51] vs. 0.46 [0.36–0.59] mg/dL, *p* = 0.044), likely reflecting greater infusion volumes. At admission, heart rate was significantly higher in the DKA group (median 118 [102–134] vs. 96 [89–111] bpm; *p* < 0.001), whereas systolic and diastolic blood pressure did not differ significantly between groups (systolic 117 [104–127] vs. 123 [111–135] mmHg, *p* = 0.106; diastolic 76 [68–87] vs. 79 [72–88] mmHg, *p* = 0.280). Weight-adjusted infusion volume during the acute treatment phase was substantially higher in the DKA group (109.5 [92.9–127.4] vs. 45.9 [30.9–63.4] mL/kg; *p* < 0.001) (Supplemental Table S1).

### Echocardiographic parameters

#### Standard echocardiographic parameters

**Fig. 4:**
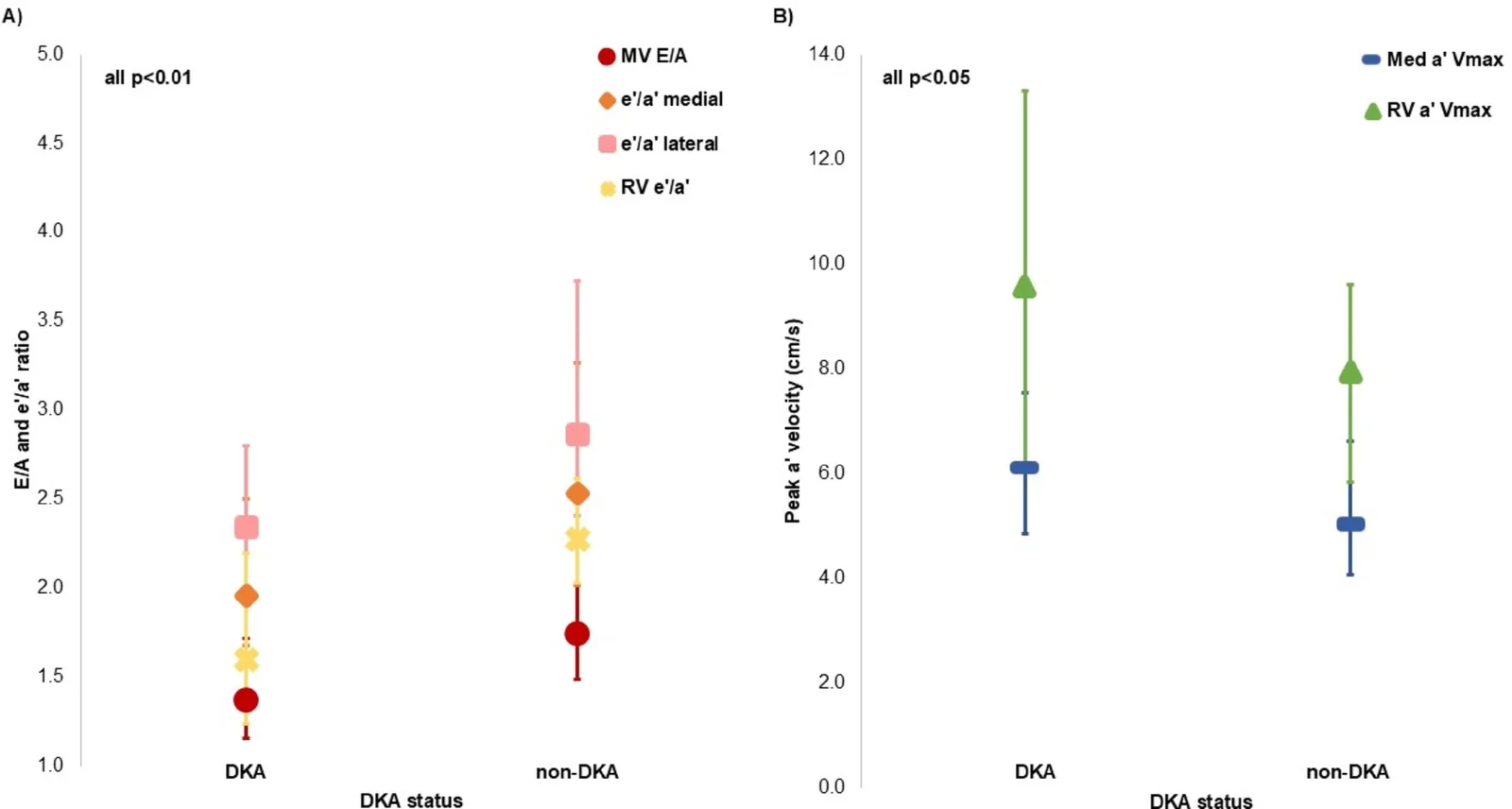
Echocardiographic parameters in DKA and non-DKA groups after 24 h. Statistically significant echocardiographic parameters are depicted for the DKA (n = 43) and non-DKA (n = 38) groups after 24 h. E/A ratios are shown on the left y-axis; peak a′-wave velocities (Med a′ Vmax, RV a′ Vmax) are plotted on the right y-axis. Each parameter is depicted by a distinct pictogram indicating the median; whiskers represent the interquartile range. Brackets show the p-values for comparison between DKA and non-DKA groups. MV E/A, mitral valve early-to-late diastolic filling ratio; e′/a′ medial, ratio of early to late diastolic tissue velocities at the septal mitral annulus; e′/a′ lateral, ratio at the lateral mitral annulus; RV e′/a′, right ventricular e′/a′ ratio; Med a′ Vmax, peak late diastolic tissue velocity at the septal mitral annulus; RV a′ Vmax, peak late diastolic velocity at the tricuspid annulus

Mitral valve E/A ratio (MV E/A), e′/a′ medial, e′/a′ lateral, and RV e′/a′ were all significantly lower in the DKA group compared with the non-DKA group (MV E/A: 1.37 [1.15–1.71] vs. 1.74 [1.49–2.55]; e′/a′ medial: 1.95 [1.67–2.50] vs. 2.53 [2.01–3.27]; e′/a′ lateral: 2.34 [1.99–2.80] vs. 2.86 [2.40–3.73]; RV e′/a′: 1.60 [1.23–2.19] vs. 2.28 [2.03–2.62]; all *p* < 0.01) (Fig. 4). MV E/A correlated significantly with pH (r = 0.433, *p* < 0.001), bicarbonate (r = 0.451, *p* < 0.001), troponin I at baseline (r = − 0.449, *p* < 0.001) and at 24 h (r = − 0.468, *p* < 0.001), as well as NT-proBNP at 24 h (r = − 0.342, *p* = 0.005). Conversely, peak a′-wave velocities—Med a′ Vmax and RV a′ Vmax—were significantly higher in the DKA group (Med a′ Vmax: 6.09 [4.84–7.54] vs. 5.03 [4.07–6.61] cm/s, p = 0.041; RV a′ Vmax: 9.57 [6.19–13.30] vs. 7.94 [5.83–9.60] cm/s, *p* = 0.031) (Fig. 4). Other parameters did not differ significantly between groups (Supplemental Table S2).

Within the DKA group, markers of diastolic function improved significantly after 6 days (MV E/A: 1.37 [1.15–1.71] at 24 h vs. 1.75 [1.43–2.07] at 6 days, *p* < 0.001; Lat e′ Vmax: 17.10 [14.80–18.80] vs. 19.10 [15.40–22.40] cm/s, *p* = 0.043; e′/a′ lateral: 2.34 [1.99–2.80] vs. 2.51 [2.05–3.58], *p* = 0.014). Structural parameters also changed: the z-score of the right ventricular end-diastolic diameter (z RVDD) decreased (1.50 [0.02–2.71] vs. 1.06 [0.11–1.96], *p* = 0.009), while the z-scores of right ventricular anterior wall thickness in diastole (z RVAWd) and left ventricular end-diastolic diameter (z LVEDD) increased significantly (z RVAWd: 0.78 [0.27–1.33] vs. 1.02 [0.47–1.33], *p* = 0.031; z LVEDD: − 0.59 [−  1.04–0.12] vs. 0.04 [−  0.67–0.64], *p* = 0.007) (Supplemental Table S2). Heart rate during the echocardiographic (E/A) assessment at 24 h (overall median 91 [IQR 75–105.5] bpm) was significantly higher in the DKA group than in the non-DKA group (*p* < 0.001, Mann–Whitney U test), mirroring the admission difference; within the DKA group it decreased significantly by 6 days (*p* < 0.001, Wilcoxon signed-rank test), in parallel with the partial normalization of the E/A ratio.

#### Strain values

Z-scores of right ventricular and left ventricular longitudinal strain in the four-chamber view (z RV A4C and z LV A4C) did not differ significantly between the DKA and non-DKA groups at 24 h. However, z LV A4C improved significantly after 6 days within the DKA group (1.72 [0.13–2.37] vs. 1.03 [−  0.07–1.73], *p* = 0.011) (Supplemental Table S2).

**Fig. 5:**
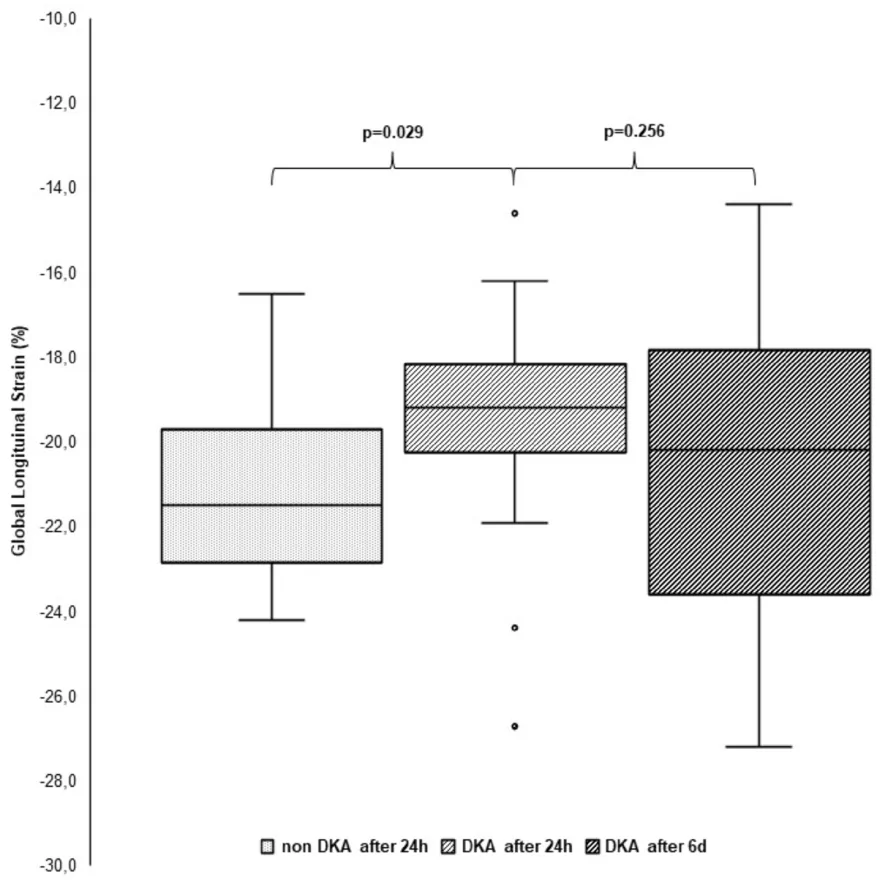
Left ventricular global longitudinal strain (GLS) by DKA status and timepoint. GLS values are shown for non-DKA patients after 24 h, and for DKA patients after 24 h and after 6 days. Boxes represent the IQR with medians indicated by horizontal lines and numerical values. Whiskers indicate range excluding outliers; dots represent outliers. Brackets indicate comparisons between groups and time points (significance threshold *p* < 0.05). Sample sizes were n = 16 (non-DKA, 24 h), n = 24 (DKA, 24 h), and n = 19 (DKA, 6 days). GLS was significantly less negative in the DKA group than in the non-DKA group at 24 h (*p* = 0.029), whereas the within-DKA comparison between 24 h and 6 days (paired Wilcoxon signed-rank test) was not significant. Less negative GLS values indicate worse systolic deformation. No 6-day measurement is available for the non-DKA group

In contrast, left ventricular GLS was significantly less negative (i.e., reduced) in the DKA group (n = 24) compared with the non-DKA group (n=16) (−  19.2 [−  20.4 to − 18.1] vs. − 21.5 [−  23.0 to − 18.9], *p* = 0.029) (Fig. 5). No significant change in GLS was observed within the DKA group between 24 h and 6 days. GLS correlated significantly with HbA1c (r = 0.395, *p* = 0.014), initial glucose (r= 0.353, *p* = 0.025), baseline creatinine (r = 0.339, *p* = 0.035), and infusion volume during the first 24 h (r = 0.500, *p* = 0.001).

## Discussion

In this prospective pediatric study of DKA at T1D onset, metabolic and subclinical cardiac disturbances were prevalent. Troponin I and NT-proBNP were elevated and correlated significantly with DKA severity. Echocardiography revealed signs of subclinical cardiac dysfunction including alterations in diastolic filling dynamics—reduced E/A ratios and elevated peak A′ velocities bilaterally—and impaired systolic deformation reflected by reduced GLS. Most values remained within the normal range, indicating subclinical involvement.

Troponin I, an indicator of cardiomyocyte damage, can be elevated in DKA even without myocardial infarction or acute coronary syndrome [3–6, 20]. In this study, troponin I elevation was associated with DKA severity, consistent with prior adult data. Several mechanisms may contribute. George et al. demonstrated elevated catecholamine levels and increased cardiac contractility in DKA [12], supporting a hyperadrenergic state that raises myocardial oxygen demand. Additionally, elevated free fatty acids in insulin-deficient states may incorporate into cardiomyocyte lipid membranes, impairing membrane integrity and promoting troponin I release [21–23]. Severe acidosis may further contribute through intracellular calcium accumulation via altered sarcoplasmic reticulum ion transport, activating proteolytic pathways and impairing calcium-contractile protein interaction, potentially causing myocardial stunning [5, 21, 24]. Over time, troponin I levels remained stable in the DKA group, neither increasing further nor declining significantly.

NT-proBNP is released by cardiomyocytes in response to increased intracardiac pressure. Its elevation in this cohort was associated with DKA severity and increased further after 24 h, with the median 24-h value exceeding the pathological cutoff. Importantly, NT-proBNP elevation did not correlate with infusion volume, arguing against myocardial wall tension from volume loading as the primary driver [25]. Metabolic acidosis can induce myocardial stress and hypoxia, which selectively upregulate B-type natriuretic peptide expression [8, 26]. The proinflammatory state in DKA—evidenced by elevated CRP in the absence of overt infection and its significant correlation with NT-proBNP—may also promote natriuretic peptide release [27–29]. Although creatinine did not suggest acute kidney injury in this cohort, potentially due to the larger infusion volumes in the DKA group [30], additional renal function markers would be required to conclusively exclude reduced renal clearance as a contributing mechanism.

Echocardiographic analysis revealed reduced E/A ratios and elevated peak a′-wave velocities in both ventricles in the DKA group, consistent with impaired early diastolic relaxation and compensatory enhanced atrial contraction. All values remained within the normal range. These findings likely reflect transient hemodynamic alterations during the acute metabolic state: after 6 days, E/A ratios partially normalized within the DKA group. Elevated heart rates are known to occur in DKA and are a recognized physiological determinant of transmitral filling [31]; heart rate recorded during echocardiography was accordingly higher in the DKA group at 24 h and fell in parallel with the E/A ratio by day 6. Because transmitral E and A velocities are not routinely corrected for heart rate—interpretation instead resting on age, rhythm, and the E/A relationship—these indices were not statistically adjusted for heart rate; the altered diastolic filling is therefore best interpreted as part of the integrated acute physiological state rather than as intrinsic diastolic dysfunction. No correlation was found between MV E/A and CRP or infusion volume, making acute inflammation and dehydration unlikely as primary drivers of the diastolic abnormalities; rather [29, 32], pH and bicarbonate correlations implicate acute acidosis. For signs of established diabetic cardiomyopathy to be present at disease onset would be highly unusual, though subclinical early myocardial changes cannot be excluded [33].

GLS was significantly reduced in the DKA group and did not normalize after 6 days, despite remaining within the normal range. In contrast to diastolic parameters, GLS did not correlate with pH or bicarbonate, but correlated significantly with glycemic status, infusion volume, and baseline creatinine, suggesting that systolic deformation is more sensitive to metabolic and hemodynamic factors than to acute acidosis. The direction of the infusion volume effect—greater volume associated with less negative (i.e., reduced) GLS—appears paradoxical relative to expected Frank-Starling preload augmentation but may reflect competing effects of volume status, myocardial stress, and early remodeling [34]. Both acute and chronic hyperglycemia have been associated with structural myocardial damage and impaired GLS in adults [35], consistent with the observed correlation between glycemic markers and GLS in this pediatric cohort.

### Strengths

To the best of our knowledge, this is the first prospective pediatric study to systematically investigate myocardial changes at T1D onset with DKA using multimodal endpoints comprising cardiac biomarkers and echocardiography with strain analysis. Data collection began at disease onset and extended beyond the acute phase. Strain analysis was performed using AutoStrain, a method that reduces intra- and interobserver variability. Age- and height-adjusted z-scores were calculated for echocardiographic parameters. These findings have potential clinical relevance for cardiovascular risk stratification in pediatric T1D.

### Limitations

This single-center study had a relatively small sample size, particularly across all four biomarker time points, limiting statistical power and subgroup analyses. The short follow-up duration precluded distinction between transient and persistent cardiac alterations. Echocardiography could not always be performed at predefined time points (planned 1 and 6 days; achieved at average 2 and 8 days), introducing temporal variability. Although ECGs were performed in all patients and showed no arrythmias, no formal ECG analyses are reported here, given the echocardiographic focus of the study. Pediatric reference values for echocardiographic parameters vary by age, BMI, and body surface area (BSA), and validated z-scores were not available for all parameters. The absence of a healthy control group and of comprehensive renal and inflammatory biomarkers further limits mechanistic interpretation. Heart rate during echocardiography was higher in the DKA group, consistent with the acute state and with the higher admission heart rate (Supplemental Table S1); the diastolic indices were not adjusted for heart rate, because transmitral and tissue-Doppler parameters are not routinely heart-rate-corrected in clinical practice—heart rate being a physiological determinant of diastolic filling—so these findings should be read as descriptors of the acute physiological state rather than intrinsic dysfunction. Furthermore, use of a hyperglycemic non-DKA group rather than healthy controls likely underestimates the true DKA effect on cardiac parameters. Multivariable models were applied to the cardiac biomarkers and identified lower bicarbonate—and, for NT-proBNP, younger age—as independent predictors; echocardiographic parameters, however, were not modeled, and infusion volume was not available as a covariate, so residual confounding of these outcomes cannot be excluded. The limited sample size also constrained the number of covariates and the precision of the adjusted estimates. Further, as a single-center study conducted at a German tertiary pediatric diabetes center, generalizability to other healthcare settings, countries with different DKA incidence rates, or ethnically diverse populations requires cautious interpretation. The proportion of participants presenting with DKA (53.1%) exceeded the ~20% typically reported for new-onset pediatric T1D; this most likely reflects the single-center tertiary referral structure, the exclusion of patients initially admitted to other hospitals, and catchment-area characteristics rather than a truly higher population incidence, and should be considered when generalizing these findings. In addition, quantitative beta-hydroxybutyrate was not measured routinely and ketonuria was not systematically recorded; although ketosis fulfilling ISPAD criteria can reasonably be assumed given the degree of hyperglycemia and acidosis, the absence of documented ketone data is a methodological limitation of this prospective study. Finally, echocardiographic and strain parameters were available for varying subsets of participants; left ventricular GLS at 24 h was evaluable in 40 of 81 participants (49.4%). Missingness was predominantly technical, reflecting image quality and the speckle-tracking feasibility. To examine whether it was also related to clinical status, we compared participants with and without evaluable GLS (Supplemental Table S5). Evaluability did not differ by DKA status (24 of 43 [55.8%] vs. 16 of 38 [42.1%]; *p* = 0.268) or sex (*p* = 0.821), and none of 17 demographic, hemodynamic, acid–base, metabolic, renal, inflammatory or cardiac-biomarker variables differed between groups, either in the whole cohort (all *p* ≥ 0.128) or within the DKA group (all *p* ≥ 0.089) (Supplemental Table S5). In an exploratory logistic regression, pH, troponin I, age and heart rate during echocardiography did not predict evaluability (model χ^2^ = 6.79, df = 4, *p* = 0.148; n = 65). A sensitivity analysis for four-chamber longitudinal strain (z LV A4C, evaluable in 59 of 81 participants) gave concordant results (evaluability by DKA status *p* = 1.000; all 17 comparisons *p* ≥ 0.137). These analyses cannot establish that data were missing completely at random, and residual selection bias cannot be excluded. Within the DKA group, participants with evaluable GLS were, if anything, slightly more acidotic (pH 7.10 vs. 7.16; bicarbonate 9.3 vs. 10.8 mmol/L; both *p* > 0.28), arguing against preferential exclusion of the most severely affected children. Neither pH nor bicarbonate was associated with GLS, making it unlikely that these imbalances explain the observed between-group difference (Supplemental Table S4).

## Conclusions

In children with T1D, DKA at onset is associated with biventricular subclinical cardiac involvement, evidenced by elevated cardiac biomarkers and altered echocardiographic parameters. Most findings remained within the normal range. Whether these changes represent transient adaptive responses to metabolic derangement or carry long-term prognostic significance remains to be determined. Given the high lifetime cardiovascular risk in people with T1D—potentially exacerbated by DKA-associated myocardial involvement at onset—acute cardiac monitoring during severe DKA may be warranted in selected patients, rather than for long-term cardiovascular risk stratification. Longitudinal studies with extended follow-up are needed to clarify the prognostic significance of these early cardiac changes.

## Supplementary Information

Below is the link to the electronic supplementary material.

Supplementary Material 1

## Data Availability

The data that support the findings of this study are available from the corresponding author upon reasonable request.

## Abbreviations

AV VTI: Aortic valve velocity–time integral
BE: Base excess
BMI: Body mass index
BMI-SDS: BMI standard deviation score
BSA: Body surface area
BPM: Beats per minute
CMIA: Chemiluminescent microparticle immunoassay
CK-MB: Creatine kinase-MB isoenzyme
CRP: C-reactive protein
DKA: Diabetic ketoacidosis
ECG: Electrocardiogram
EF: Ejection fraction
E/A: Early-to-late diastolic filling ratio
e′: Early diastolic tissue velocity
a′: Late diastolic tissue velocity (atrial)
FAC LV: Fractional area change of the left ventricle
GLS: Global longitudinal strain
HbA1c: Glycated hemoglobin
IQR: Interquartile range
ISPAD: International Society for Pediatric and Adolescent Diabetes
IVSd/IVSs: Interventricular septal thickness in diastole/systole
Lat A′/E′ Vmax: Lateral a′/e′-peak velocity
LVEDD/LVESD: Left ventricular end-diastolic/end-systolic diameter
LVPWd/LVPWs: Left ventricular posterior wall thickness in diastole/systole
LV A4C: Left ventricular longitudinal strain in the four-chamber view
MAPSE: Mitral annular plane systolic excursion
Med a′/e′/s′ Vmax: Medial a′/e′/s′-peak velocity
MV E/A: Mitral valve E/A ratio
NT-proBNP: N-terminal pro-B-type natriuretic peptide
RV A4C: Right ventricular longitudinal strain in the four-chamber view
RV a′/e′/s′ Vmax: Right ventricular a′/e′/s′-peak velocity
RV e′/a′: Right ventricular e′/a′ ratio
RVAWd: Right ventricular anterior wall thickness in diastole
RVDD: Right ventricular end-diastolic diameter
TAPSE: Tricuspid annular plane systolic excursion
T1D: Type 1 diabetes
TV E/A: Tricuspid valve E/A ratio
