# Supplementary material for: Subclinical cardiac involvement during diabetic ketoacidosis at type 1 diabetes onset in youth: biomarker elevation, altered diastolic filling, and reduced global longitudinal strain: a prospective cohort study
Source: Cardiovasc Diabetol. 2026 Sep 18;25:283. doi: 10.1186/s12933-026-03377-9 (PMC13591842; doi:10.1186/s12933-026-03377-9)
Supplement: Supplementary file 1 [file 12933_2026_3377_MOESM1_ESM.docx]

**Supplemental Material**

**Supplemental Table S1 — Baseline Clinical and Laboratory Characteristics of Participants with and without Diabetic Ketoacidosis**

|  | **Overall  (n=81)** | **DKA group (n=43)** | **Non-DKA group (n=38)** | **p value** |
| --- | --- | --- | --- | --- |
| **Demographics** |  |  |  |  |
| Male sex, n (%) | 49  (60.5) | 27  (62.8) | 22  (57.9) | 0.820 |
| Age (years) | 9.8  (5.2–14.1) | 9.8  (3.2–13.9) | 9.8  (5.9–14.6) | 0.188 |
| Weight (kg) | 30.0  (18.0–48.5) | 29.0  (14.0–44.0) | 37.5  (20.6–53.3) | 0.087 |
| BMI (kg/m²) | 15.5  (14.3–18.1) | 15.4  (14.2–17.6) | 15.5  (14.6–18.4) | 0.283 |
| BMI-SDS | −0.67  (−1.37 - 0.00) | −0.79  (−1.84 - 0.09) | −0.63  (−1.17 - −0.01) | 0.311 |
| **Vital signs and fluid therapy at admission** |  |  |  |  |
| Systolic blood pressure (mmHg) | 120 (107–131) | 117 (104–127) | 123 (111–135) | 0.106 |
| Diastolic blood pressure (mmHg) | 78 (70–87) | 76 (68–87) | 79 (72–88) | 0.280 |
| Heart rate (bpm) | 109 (93–126) | 118 (102–134) | 96 (89–111) | <0.001 |
| Infusion volume (mL/kg)ᵗ | 89.1 (47.6–112.3) | 109.5 (92.9–127.4) | 45.9 (30.9–63.4) | <0.001 |
| **Immunological characteristics** |  |  |  |  |
| Islet autoantibody-positive, n (%) | 76 (93.8) | 42 (97.7) | 34 (89.5) | 0.181ᵘ |
| **Acid-base and metabolic parameters** |  |  |  |  |
| pH | 7.31  (7.10–7.40) | 7.12  (7.03–7.23) | 7.41  (7.36–7.42) | <0.001 |
| Bicarbonate (mmol/L) | 15.9  (9.5–23.6)ᵃ | 10.0  (7.5–12.9)ᵃ | 23.7  (22.2–24.7) | <0.001 |
| Base excess (mmol/L) | −11.6  (−21.0 - −1.1)ᵃ | −20.4  (−25.3–−16.2)ᵃ | −1.0  (−2.7–0.2) | <0.001 |
| pCO₂ (mmHg) | 30.6  (19.8–37.3)ᵇ | 20.3  (16.4–28.2)ᵃ | 37.6  (33.4–41.4)ᶜ | <0.001 |
| Osmolality (mOsm/kg) | 296.6  (292.2–300.6)ᵈ | 297.7  (293.0–305.2)ᵉ | 295.5  (291.7–299.5) | 0.117 |
| **Glycemic parameters** |  |  |  |  |
| HbA1c (% / mmol/mol) | 12.2  (11.2–13.8) /  110  (100–128)ᵇ | 12.3  (11.5–14.1) / 111  (102–131)ᶠ | 12.2  (10.1–13.5) /  110  (87–124) | 0.263 |
| Glucose (mg/dL / mmol/L) | 421  (327–540) /  23.4  (18.1–30.0) | 473  (370–614) / 26.3  (20.5–34.1) | 395  (299–532) /  21.9  (16.6–29.5) | 0.047 |
| **Laboratory parameters** |  |  |  |  |
| Creatinine (mg/dL) | 0.54  (0.37–0.65)ᵍ | 0.57  (0.37–0.72)ᵃ | 0.48  (0.37–0.61)ʰ | 0.186 |
| CRP (mg/dL) | 0.14  (0.07–0.37)ⁱ | 0.29  (0.08–0.80) | 0.12  (0.06–0.17)ʰ | 0.014 |
| **Cardiac biomarkers (baseline)** |  |  |  |  |
| Troponin I (ng/L) | 4.2  (3.9-8.1) | 5.7  (4.1–10.8) | 3.9  (3.9–4.6) | <0.001 |
| NT-proBNP (ng/L) | 63.0 (24.5-121.0) | 87.0  (41.0–231.0) | 40.0  (16.8–69.0) | 0.002 |

Data are presented as median (IQR) or n (%). BMI, body mass index; BMI-SDS, BMI standard deviation score; CRP, C-reactive protein; HbA1c, glycated hemoglobin; IQR, interquartile range; NT-proBNP, N-terminal pro-B-type natriuretic peptide. Superscripts indicate missing values: ᵃn=42, ᵇn=79, ᶜn=37, ᵈn=77, ᵉn=39, ᶠn=41, ᵍn=78, ʰn=36, ⁱn=79. Group comparisons by Mann-Whitney U test (continuous) or chi-square test (categorical). p values <0.05 are considered statistically significant. Vital signs and infusion volume were available in a subset of participants: systolic blood pressure n=72 (DKA 39/non-DKA 33), diastolic blood pressure n=73 (40/33), heart rate n=74 (41/33), and weight-adjusted infusion volume n=71 (40/31). ᵗInfusion volume denotes cumulative weight-adjusted fluid administered during the acute treatment phase in the first 24 hours. Islet autoantibody status was available for all 81 participants; among the 76 antibody-positive participants the median number of positive autoantibodies was 3 (IQR 2–4) and did not differ between groups (p=0.621, Mann–Whitney U test). ᵘFisher’s exact test.

**Supplemental Table S2 — Echocardiographic Parameters at 24 Hours and 6 Days in Participants with and without Diabetic Ketoacidosis**

| **Parameter** | **Non-DKA 24 h  (n=38)** | **DKA 24 h (n=43)** | **p value DKA vs. non-DKA** | **DKA 6 days (n=43)** | **p value DKA 24 h vs. 6 days** |
| --- | --- | --- | --- | --- | --- |
| **General systolic function** |  |  |  |  |  |
| Simpson EF (%) | 61.0  (58.6–63.8)ᵃ | 61.0  (56.9–64.2)ᵇ | NS | 62.3  (57.1–65.1)ᶜ | NS |
| FAC LV (%) | 57.0  (51.0–59.9)ᵃ | 53.0  (49.7–59.2)ᵈ | NS | 55.0  (50.5–59.9)ᵉ | NS |
| Bullet LV EF (%) | 63.7  (56.3–68.0)ᶠ | 60.0  (54.7–64.1)ᵍ | NS | 59.4  (57.0–63.2)ᶠ | NS |
| EF Teichholz (%) | 62.1  (58.2–68.1)ʰ | 65.2  (58.4–68.8)ⁱ | NS | 65.5  (60.6–69.9)ʲ | NS |
| AV VTI (cm) | 21.0  (17.1–25.9)ʰ | 20.7  (17.5–23.5)ⁱ | NS | 22.9  (20.2–25.3)ᵏ | 0.008 |
| TAPSE (cm) | 2.01  (1.9–2.4)ˡ | 1.90  (1.8–2.2)ⁱ | NS | 2.19  (1.90–2.40)ᵐ | 0.016 |
| MAPSE (cm) | 1.50  (1.2–1.8)ˡ | 1.50  (1.3–1.6)ⁿ | NS | 1.53  (1.36–1.67)ᵐ | NS |
| **Systolic peak velocities** |  |  |  |  |  |
| Med s' Vmax (cm/s) | 7.30  (6.2–8.1)ˡ | 7.83  (6.5–9.0)ⁱ | NS | 8.15  (7.50–9.33)ᵐ | NS |
| Lat s' Vmax (cm/s) | 9.15  (7.3–12.5)ˡ | 10.10  (8.0–13.4)ⁱ | NS | 9.36 (8.65–11.40)° | NS |
| RV s' Vmax (cm/s) | 13.60  (11.8–14.5)ᵖ | 12.80 (12.0–14.4)ᵠ | NS | 14.45 (12.58–16.38)ʳ | NS |
| **Diastolic parameters** |  |  |  |  |  |
| MV E/A | 1.74  (1.5–2.6)ˢ | 1.37  (1.2–1.7)ⁱ | 0.002 | 1.75  (1.43–2.07)ᵗ | <0.001 |
| TV E/A | 1.9  (1.4–2.2)ᵃ | 1.5  (1.2–1.9)ᵠ | NS | 1.6  (1.3–2.0)ᵛ | NS |
| Med e' Vmax (cm/s) | 13.20  (11.4–15.5)ᵘ | 12.80  (11.5–14.1)ᵍ | NS | 13.70  (12.5–15.3)ᵏ | NS |
| Med a' Vmax (cm/s) | 5.03  (4.1–6.6)ˡ | 6.09  (4.8–7.5)ⁱ | 0.041 | 6.54  (5.4–7.3)ᵐ | NS |
| e'/a' medial | 2.53  (2.0–3.3)ᵘ | 1.95  (1.7–2.5)ⁱ | 0.006 | 2.12  (1.7–2.6)ᵐ | NS |
| E/e′ medial | 6.38  (5.66–8.03) | 6.81  (5.96–7.48) | NS | 6.98  (6.25–8.29) | NS |
| Lat e' Vmax (cm/s) | 17.20  (15.5–22.1)ˡ | 17.10  (14.8–18.8)ⁱ | NS | 19.10  (15.4–22.4)° | 0.043 |
| Lat a' Vmax (cm/s) | 6.37  (5.1–7.4)ˡ | 6.80  (5.8–7.8)ⁱ | NS | 6.49  (6.0–9.0)° | NS |
| e'/a' lateral | 2.86  (2.4–3.7)ˡ | 2.34  (2.0–2.8)ⁱ | 0.001 | 2.51  (2.1–3.6)° | 0.014 |
| E/e′ lateral | 4.91  (4.24–5.64) | 5.23  (4.55–5.91) | NS | 5.25  (4.22–6.57) | NS |
| E/e′ mean | 5.40  (5.10–6.20) | 6.20  (5.73–6.95) | 0.035 | 6.90  (5.30–7.75) | NS |
| RV e' Vmax (cm/s) | 16.90  (14.3–18.8)ᵖ | 15.80  (13.4–16.6)ᵠ | NS | 16.50  (14.6–19.5)ʳ | NS |
| RV a' Vmax (cm/s) | 7.94  (5.8–9.6)ᵖ | 9.57  (6.2–13.3)ᵠ | 0.031 | 9.85  (7.9–11.2)ʳ | NS |
| RV e'/a' | 2.28  (2.0–2.6)ᵖ | 1.60  (1.2–2.2)ᵠ | 0.002 | 1.90  (1.5–2.1)ʳ | NS |
| **Ventricular dimensions and wall thickness (z-scores)** |  |  |  |  |  |
| z RVAWd | 0.95  (0.72–1.55)ᵃ | 0.78  (0.27–1.33)ᵈ | NS | 1.02  (0.47–1.33)ᵉ | 0.031 |
| z RVDD | 1.78  (−0.25–2.73)ᵃ | 1.50  (0.02–2.71)ᵈ | NS | 1.06  (0.11–1.96)ᵉ | 0.009 |
| z IVSd | −0.73  (−1.23–0.44)ʰ | −0.59  (−1.21–0.39)ⁱ | NS | −0.59  (−1.34–0.23)ʲ | NS |
| z IVSs | −0.27  (−0.69–0.41)ᵘ | −0.37  (−0.71–0.27)ⁿ | NS | −0.20  (−0.45–0.36)ᵏ | NS |
| z LVEDD | −0.23  (−1.51–0.28)ʰ | −0.59  (−1.04–0.12)ⁱ | NS | 0.04  (−0.67–0.64)ʲ | 0.007 |
| z LVESD | 0.02  (−0.96–0.91)ʰ | −0.11  (−0.78–0.54)ⁱ | NS | 0.25  (−0.73–0.94)ʲ | NS |
| z LVPWd | −0.30  (−1.01–0.65)ʰ | −0.29  (−0.94–0.69)ⁱ | NS | −0.44  (−0.77–0.65)ʲ | NS |
| z LVPWs | −0.16  (−0.55–0.34)ᵘ | −0.30  (−0.82–0.53)ⁿ | NS | −0.02  (−0.44–0.39)ᵐ | NS |
| **Speckle-tracking strain (AutoStrain)** |  |  |  |  |  |
| z RV A4C | 1.28  (0.42–1.86)ᵉ | 1.24  (0.03–1.68)ᵠ | NS | 0.50  (−0.90–1.59)ˢ | NS |
| z LV A4C | 0.85  (0.29–1.81)ˢ | 1.72  (0.13–2.37)ᵠ | NS | 1.03  (−0.07–1.73)ˢ | 0.011 |
| GLS LV (%) | −21.5  (−23.0–−18.9)ʷ | −19.2  (−20.4–−18.1)ˣ | 0.029 | −20.2  (−23.6–−17.7)ʸ | NS |

Data are presented as median (IQR). Superscripts indicate deviating sample sizes: ᵃn=20, ᵇn=32, ᶜn=26, ᵈn=29, ᵉn=25, ᶠn=31, ᵍn=37, ʰn=33, ⁱn=35, ʲn=31, ᵏn=30, ˡn=32, ᵐn=28, ⁿn=34, °n=29, ᵖn=29 (non-DKA), ᵠn=31, ʳn=26, ˢn=16/28 (non-DKA/DKA 24h), ᵗn=29, ᵘn=31, ᵛn=26, ʷn=16, ˣn=24, ʸn=19. Sample sizes for the E/e′ ratios are given here rather than by superscript: medial E/e′ n=31 (non-DKA 24 h), 37 (DKA 24 h) and 28 (DKA 6 days); lateral E/e′ n=32, 35 and 29; mean E/e′ n=23, 22 and 18, respectively. AV VTI, aortic valve velocity-time integral; E/e′, ratio of early transmitral inflow velocity (E) to early diastolic mitral annular velocity (e′), with mean E/e′ denoting the average of the medial and lateral ratio; EF, ejection fraction; FAC LV, fractional area change left ventricle; GLS, global longitudinal strain (by speckle-tracking echocardiography using AutoStrain; more negative values indicate better systolic function); IQR, interquartile range; IVSd/IVSs, interventricular septal thickness in diastole/systole; Lat A'/E'/S' Vmax, lateral A'/E'/S'-peak velocity; LVEDD/LVESD, left ventricular end-diastolic/end-systolic diameter; LVPWd/LVPWs, left ventricular posterior wall thickness in diastole/systole; MAPSE, mitral annular plane systolic excursion; Med A'/E'/S' Vmax, medial A'/E'/S'-peak velocity; MV E/A, mitral valve E/A ratio; NS, not significant; RV A4C, right ventricular longitudinal strain four-chamber view; RV A'/E'/S' Vmax, right ventricular A'/E'/S'-peak velocity; RVAWd, right ventricular anterior wall thickness in diastole; RVDD, right ventricular end-diastolic diameter; TAPSE, tricuspid annular plane systolic excursion; TV E/A, tricuspid valve E/A ratio; z LV A4C, z-score left ventricular longitudinal strain four-chamber view; z LV/RVAWd/DD, z-scores ventricular dimensions. Group comparison by Mann-Whitney U test; within-DKA comparison by Wilcoxon signed-rank test. For the E/e′ ratios the within-DKA comparison was based on 27 (medial), 27 (lateral) and 15 (mean) complete 24-hour/6-day pairs. p <0.05 considered statistically significant.

**Supplemental Table S3 — Multivariable Linear Regression Models for Log-Transformed Baseline Troponin I and NT-proBNP**

| **Predictor** | **B** | **95% CI** | **β** | **p** |
| --- | --- | --- | --- | --- |
| Log troponin I — model R² = 0.35 (p < 0.001) |  |  |  |  |
| Age | −0.021 | −0.048 to 0.007 | −0.17 | 0.139 |
| HbA1c | −0.062 | −0.142 to 0.018 | −0.18 | 0.126 |
| Osmolality | 0.009 | −0.001 to 0.020 | 0.19 | 0.086 |
| Glucose | −0.001 | −0.002 to 0.000 | −0.19 | 0.090 |
| Bicarbonate | −0.047 | −0.068 to −0.027 | −0.51 | <0.001 |
| Log NT-proBNP — model R² = 0.48 (p < 0.001) |  |  |  |  |
| Age | −0.087 | −0.129 to −0.045 | −0.43 | <0.001 |
| HbA1c | −0.117 | −0.238 to 0.004 | −0.20 | 0.057 |
| Osmolality | 0.006 | −0.010 to 0.022 | 0.08 | 0.433 |
| Glucose | 0.001 | 0.000 to 0.002 | 0.13 | 0.180 |
| Bicarbonate | −0.067 | −0.098 to −0.035 | −0.43 | <0.001 |

Prespecified multivariable linear regression with natural log-transformed baseline troponin I and N-terminal pro-B-type natriuretic peptide (NT-proBNP) as dependent variables (n=65; four cases excluded listwise for missing osmolality). B, unstandardized regression coefficient per unit (age in years, HbA1c in %, serum osmolality in mosmol/kg, glucose in mg/dL, bicarbonate in mmol/L); β, standardized coefficient; CI, confidence interval; p, two-sided significance. Troponin I: R² = 0.35, F(5,59) = 6.43, p < 0.001; NT-proBNP: R² = 0.48, F(5,59) = 10.73, p < 0.001. All variance inflation factors were < 1.3. Creatinine was excluded a priori because it is age- and muscle-mass-dependent, was not independently associated with either biomarker, and increased multicollinearity. Excluding one influential case with extreme serum osmolality (385.1 mosmol/kg; Cook’s distance > 1) left the bicarbonate associations essentially unchanged (troponin I β = −0.43; NT-proBNP β = −0.39; both p < 0.001).

**Supplemental Table S4 —** Univariable Linear Regression of Log-Transformed Baseline Troponin I and NT-proBNP, Medial and Lateral E/e′, and Left Ventricular Global Longitudinal Strain on Demographic, Metabolic, and Laboratory Parameters

| **Predictor** | **n** | **B** | **95% CI** | **β** | **p** |
| --- | --- | --- | --- | --- | --- |
| **Log troponin I – univariable models** |  |  |  |  |  |
| Age | 66 | −0.032 | −0.062 to −0.003 | −0.27 | 0.032 |
| Weight | 66 | −0.005 | −0.013 to 0.002 | −0.18 | 0.149 |
| BMI-SDS | 66 | 0.016 | −0.104 to 0.136 | 0.03 | 0.789 |
| pH | 66 | −1.700 | −2.537 to −0.863 | −0.45 | <0.001 |
| Bicarbonate | 66 | −0.042 | −0.062 to −0.021 | −0.45 | <0.001 |
| Base excess | 66 | −0.027 | −0.040 to −0.013 | −0.43 | <0.001 |
| pCO₂ | 65 | −0.019 | −0.034 to −0.004 | −0.31 | 0.013 |
| Osmolality | 66 | 0.014 | −0.005 to 0.033 | 0.18 | 0.156 |
| HbA1c | 64 | −0.037 | −0.128 to 0.054 | −0.10 | 0.423 |
| Glucose | 66 | −0.001 | −0.002 to 0.000 | −0.15 | 0.227 |
| Creatinine | 65 | 0.137 | −0.682 to 0.956 | 0.04 | 0.740 |
| CRP | 65 | 0.005 | −0.128 to 0.138 | 0.01 | 0.938 |
| **Log NT-proBNP – univariable models** |  |  |  |  |  |
| Age | 66 | −0.108 | −0.152 to −0.064 | −0.53 | <0.001 |
| Weight | 66 | −0.027 | −0.037 to −0.017 | −0.54 | <0.001 |
| BMI-SDS | 66 | −0.058 | −0.260 to 0.144 | −0.07 | 0.568 |
| pH | 66 | −2.706 | −4.133 to −1.279 | −0.43 | <0.001 |
| Bicarbonate | 66 | −0.068 | −0.103 to −0.033 | −0.44 | <0.001 |
| Base excess | 66 | −0.044 | −0.067 to −0.020 | −0.42 | <0.001 |
| pCO₂ | 65 | −0.035 | −0.059 to −0.010 | −0.34 | 0.006 |
| Osmolality | 66 | 0.012 | −0.021 to 0.045 | 0.09 | 0.474 |
| HbA1c | 64 | −0.068 | −0.220 to 0.084 | −0.11 | 0.374 |
| Glucose | 66 | 0.001 | −0.001 to 0.003 | 0.12 | 0.329 |
| Creatinine | 65 | −0.652 | −2.013 to 0.708 | −0.12 | 0.342 |
| CRP | 65 | 0.195 | −0.024 to 0.414 | 0.22 | 0.081 |
| **Medial E/e′ (24 h) – univariable models** |  |  |  |  |  |
| Age | 63 | −0.019 | −0.095 to 0.057 | −0.06 | 0.618 |
| Weight | 63 | −0.004 | −0.021 to 0.013 | −0.06 | 0.664 |
| BMI-SDS | 63 | −0.010 | −0.267 to 0.248 | −0.01 | 0.941 |
| pH | 63 | −1.071 | −3.199 to 1.057 | −0.13 | 0.318 |
| Bicarbonate | 63 | −0.016 | −0.068 to 0.035 | −0.08 | 0.530 |
| Base excess | 63 | −0.013 | −0.048 to 0.021 | −0.10 | 0.441 |
| pCO₂ | 62 | −0.010 | −0.044 to 0.024 | −0.08 | 0.556 |
| Osmolality | 63 | −0.018 | −0.059 to 0.023 | −0.11 | 0.385 |
| HbA1c | 61 | 0.054 | −0.135 to 0.242 | 0.07 | 0.572 |
| Glucose | 63 | 0.001 | −0.001 to 0.004 | 0.14 | 0.272 |
| Creatinine | 62 | −0.848 | −2.750 to 1.054 | −0.11 | 0.376 |
| CRP | 61 | 0.045 | −0.267 to 0.357 | 0.04 | 0.773 |
| **Lateral E/e′ (24 h) – univariable models** |  |  |  |  |  |
| Age | 61 | −0.040 | −0.104 to 0.024 | −0.16 | 0.216 |
| Weight | 61 | −0.005 | −0.019 to 0.010 | −0.08 | 0.526 |
| BMI-SDS | 61 | −0.065 | −0.287 to 0.156 | −0.08 | 0.557 |
| pH | 61 | −0.385 | −2.214 to 1.444 | −0.06 | 0.675 |
| Bicarbonate | 61 | −0.019 | −0.063 to 0.025 | −0.11 | 0.388 |
| Base excess | 61 | −0.012 | −0.041 to 0.018 | −0.10 | 0.430 |
| pCO₂ | 60 | −0.015 | −0.044 to 0.013 | −0.14 | 0.279 |
| Osmolality | 61 | −0.004 | −0.040 to 0.032 | −0.03 | 0.809 |
| HbA1c | 59 | 0.036 | −0.126 to 0.199 | 0.06 | 0.656 |
| Glucose | 61 | 0.002 | 0.000 to 0.004 | 0.22 | 0.083 |
| Creatinine | 60 | −0.674 | −2.519 to 1.171 | −0.10 | 0.467 |
| CRP | 59 | 0.172 | −0.093 to 0.437 | 0.17 | 0.199 |
| **GLS (24 h) – univariable models** |  |  |  |  |  |
| Age | 35 | 0.130 | −0.048 to 0.307 | 0.25 | 0.147 |
| Weight | 35 | 0.024 | −0.029 to 0.077 | 0.16 | 0.364 |
| BMI-SDS | 35 | −0.575 | −1.310 to 0.161 | −0.27 | 0.122 |
| pH | 35 | −2.741 | −7.661 to 2.179 | −0.19 | 0.265 |
| Bicarbonate | 35 | −0.086 | −0.207 to 0.035 | −0.25 | 0.156 |
| Base excess | 35 | −0.055 | −0.134 to 0.025 | −0.24 | 0.173 |
| pCO₂ | 34 | −0.051 | −0.131 to 0.028 | −0.23 | 0.196 |
| Osmolality | 35 | 0.034 | −0.065 to 0.133 | 0.12 | 0.488 |
| HbA1c | 33 | 0.509 | 0.088 to 0.930 | 0.41 | 0.019 |
| Glucose | 35 | 0.005 | 0.000 to 0.011 | 0.33 | 0.056 |
| Creatinine | 35 | 6.583 | 1.673 to 11.492 | 0.43 | 0.010 |
| CRP | 35 | 0.714 | −0.347 to 1.776 | 0.23 | 0.180 |

Each row is a separate simple (univariable) linear regression with a single cardiac parameter as the dependent variable and one predictor. Dependent variables were natural log-transformed baseline troponin I, natural log-transformed baseline N-terminal pro-B-type natriuretic peptide (NT-proBNP), the medial (septal) and lateral mitral E/e′ ratios at 24 hours, and left ventricular global longitudinal strain (GLS) at 24 hours. B, unstandardized regression coefficient per unit (age in years, weight in kg, BMI-SDS dimensionless, pH in units, bicarbonate and base excess in mmol/L, pCO₂ in mmHg, osmolality in mosmol/kg, HbA1c in %, glucose in mg/dL, creatinine in mg/dL, CRP in mg/dL); β, standardized coefficient (equal to Pearson’s r in a single-predictor model); CI, confidence interval; n, complete cases per model. Consistent with Supplemental Table S3, one influential participant with extreme serum osmolality (385.1 mosmol/kg) was excluded a priori from all models; the remaining variation in n reflects item-level missing data. p<0.05 (two-sided) considered statistically significant. For the echocardiographic outcomes, medial and lateral E/e′ and GLS were assessed at the 24-hour time point; the smaller and differing sample sizes (medial E/e′, n=61–63; lateral E/e′, n=59–61; GLS, n=33–35) reflect the availability of tissue-Doppler and speckle-tracking measurements together with item-level missing data. GLS is expressed in % (more negative values indicate greater deformation), so a positive regression coefficient denotes less negative strain (i.e., worse systolic deformation). Univariable associations of GLS are additionally reported as Spearman correlations in the main text.

**Supplemental Table S5 — Comparison of Participants with and without Evaluable Left Ventricular Global Longitudinal Strain at 24 Hours**

| **Parameter** | **n (evaluable / not evaluable)** | **GLS evaluable** | **GLS not evaluable** | **p value** |
| --- | --- | --- | --- | --- |
| **Whole cohort (n=81)** | | | | |
| Male sex, n (%) | 40/41 | 25 (62.5) | 24 (58.5) | 0.821 |
| DKA at onset, n (%) | 40/41 | 24 (60.0) | 19 (46.3) | 0.268 |
| Age (years) | 40/41 | 9.8 (5.1–13.9) | 9.8 (5.2–14.4) | 0.727 |
| Weight (kg) | 40/41 | 30.0 (19.5–49.1) | 33.5 (17.0–48.5) | 0.895 |
| BMI-SDS | 40/41 | −0.85 (−1.35 to −0.07) | −0.49 (−1.54 to 0.11) | 0.243 |
| Heart rate at admission (bpm) | 38/36 | 111 (93–129) | 107 (92–125) | 0.978 |
| Heart rate at echocardiography, 24 h (bpm) | 40/37 | 90 (74–109) | 95 (78–106) | 0.838 |
| pH | 40/41 | 7.23 (7.07–7.40) | 7.33 (7.17–7.41) | 0.183 |
| Bicarbonate (mmol/L) | 40/40 | 13.0 (8.4–23.3) | 18.8 (11.3–24.0) | 0.128 |
| Base excess (mmol/L) | 40/40 | −15.6 (−23.9 to −1.5) | −7.1 (−19.3 to −0.6) | 0.137 |
| pCO₂ (mmHg) | 39/40 | 28.0 (18.7–36.4) | 32.9 (23.1–37.8) | 0.173 |
| Osmolality (mOsm/kg) | 36/41 | 296.3 (291.6–300.7) | 296.8 (294.0–301.7) | 0.388 |
| HbA1c (%) | 38/41 | 12.1 (10.9–13.9) | 12.5 (11.6–13.9) | 0.432 |
| Glucose (mg/dL) | 40/41 | 415 (299–512) | 451 (345–547) | 0.441 |
| Creatinine (mg/dL) | 39/39 | 0.53 (0.37–0.61) | 0.54 (0.39–0.70) | 0.522 |
| CRP (mg/dL) | 40/39 | 0.17 (0.07–0.45) | 0.13 (0.07–0.33) | 0.691 |
| Troponin I (ng/L) | 33/36 | 4.3 (3.9–7.7) | 4.2 (3.9–8.2) | 0.960 |
| NT-proBNP (ng/L) | 33/36 | 49.0 (25.5–148.5) | 63.5 (23.3–116.0) | 0.665 |
| Infusion volume, first 24 h (mL/kg) | 36/35 | 85.7 (40.8–112.1) | 89.9 (53.8–122.1) | 0.550 |
| **DKA group only (n=43)** | | | | |
| Age (years) | 24/19 | 10.8 (3.1–13.5) | 9.4 (3.4–14.3) | 0.980 |
| Weight (kg) | 24/19 | 29.3 (15.0–43.1) | 29.0 (13.0–44.8) | 0.751 |
| BMI-SDS | 24/19 | −1.10 (−1.90 to −0.41) | −0.37 (−1.61 to 0.27) | 0.089 |
| Heart rate at admission (bpm) | 23/18 | 116 (99–132) | 124 (103–137) | 0.590 |
| Heart rate at echocardiography, 24 h (bpm) | 24/17 | 105 (81–117) | 97 (88–121) | 0.801 |
| pH | 24/19 | 7.10 (7.00–7.22) | 7.16 (7.06–7.26) | 0.304 |
| Bicarbonate (mmol/L) | 24/18 | 9.3 (7.2–11.8) | 10.8 (7.8–14.2) | 0.280 |
| Base excess (mmol/L) | 24/18 | −21.9 (−25.9 to −17.9) | −20.1 (−25.0 to −13.9) | 0.303 |
| pCO₂ (mmHg) | 24/18 | 20.0 (16.7–27.2) | 22.9 (15.8–30.7) | 0.416 |
| Osmolality (mOsm/kg) | 20/19 | 297.2 (292.0–300.7) | 298.3 (295.0–307.6) | 0.160 |
| HbA1c (%) | 22/19 | 12.9 (11.4–14.5) | 12.0 (11.5–13.2) | 0.340 |
| Glucose (mg/dL) | 24/19 | 479 (368–557) | 451 (370–638) | 0.826 |
| Creatinine (mg/dL) | 23/19 | 0.56 (0.35–0.62) | 0.60 (0.39–0.86) | 0.350 |
| CRP (mg/dL) | 24/19 | 0.29 (0.07–0.72) | 0.28 (0.08–1.09) | 0.493 |
| Troponin I (ng/L) | 18/17 | 5.4 (4.0–10.4) | 7.7 (4.2–12.5) | 0.476 |
| NT-proBNP (ng/L) | 18/17 | 88.5 (37.0–277.0) | 87.0 (32.5–154.0) | 0.766 |
| Infusion volume, first 24 h (mL/kg) | 21/19 | 104.1 (92.1–121.7) | 117.0 (100.6–131.9) | 0.350 |

Data are presented as median (IQR) or n (%). Global longitudinal strain (GLS) at 24 hours was evaluable in 40 of 81 participants (49.4%). The column “n” gives the number of participants with valid data for the respective parameter in the evaluable and non-evaluable subgroups. Continuous variables were compared by Mann-Whitney U test with pairwise deletion (medians and quartiles from the same pairwise samples); categorical variables by Fisher’s exact test. BMI-SDS, body mass index standard deviation score; CRP, C-reactive protein; DKA, diabetic ketoacidosis; GLS, global longitudinal strain; HbA1c, glycated hemoglobin; IQR, interquartile range; NT-proBNP, N-terminal pro-B-type natriuretic peptide. p<0.05 (two-sided) considered statistically significant; no comparison reached this threshold. In an exploratory logistic regression of GLS evaluability on pH, troponin I, age and heart rate during echocardiography (n=65), the overall model was not significant (χ²=6.79, df=4, p=0.148) and no individual predictor reached significance. In a sensitivity analysis applying the same approach to left ventricular longitudinal strain in the four-chamber view (z LV A4C, evaluable in 59 of 81 participants [72.8%]), evaluability likewise did not differ between the DKA and non-DKA groups (31 of 43 [72.1%] vs. 28 of 38 [73.7%]; p=1.000), and none of the 17 parameters listed above differed between participants with and without evaluable z LV A4C (all p≥0.137).
